# Memory for Multiple Cache Locations and Prey Quantities in a Food-Hoarding Songbird

**DOI:** 10.3389/fpsyg.2012.00584

**Published:** 2012-12-31

**Authors:** Nicola Armstrong, Alexis Garland, K. C. Burns

**Affiliations:** ^1^School of Biological Sciences, Victoria University of WellingtonWellington, New Zealand

**Keywords:** New Zealand robin, number, field experiment, cache, memory

## Abstract

Most animals can discriminate between pairs of numbers that are each less than four without training. However, North Island robins (*Petroica longipes*), a food-hoarding songbird endemic to New Zealand, can discriminate between quantities of items as high as eight without training. Here we investigate whether robins are capable of other complex quantity discrimination tasks. We test whether their ability to discriminate between small quantities declines with (1) the number of cache sites containing prey rewards and (2) the length of time separating cache creation and retrieval (retention interval). Results showed that subjects generally performed above-chance expectations. They were equally able to discriminate between different combinations of prey quantities that were hidden from view in 2, 3, and 4 cache sites from between 1, 10, and 60 s. Overall results indicate that North Island robins can process complex quantity information involving more than two discrete quantities of items for up to 1 min long retention intervals without training.

## Introduction

Numerical processing has been demonstrated in a wide range of animal species (for a review see Brannon, 2005; Reznikova, 2007; Reznikova and Ryabko, 2011) from beetles (Carazo et al., 2009) to bears (Vonk and Beran, 2012). Small numbers of less than four are dealt with innately by most non-human species (Trick and Pylyshyn, 1994; Hauser et al., 2000; Feigenson et al., 2004). Larger number discriminations and advanced numerical processing have been shown in some highly trained lab animals (Beran, 2001; Pepperberg, 2006; Tomonaga, 2008), but also appear to be displayed naturally to some extent by wild or free-ranging animals in the absence of training (Lyon, 2003; Hunt et al., 2008; Low et al., 2009; White et al., 2009; Garland et al., 2012).

Food-hoarding animals may provide a unique window into the evolution of numerical competency in animals. Successful food-hoarding often requires accurate memory of the number of cache sites an animal has created, the number of items in each site, whether some or all of those items have been retrieved, and in some cases how long the items have been stored. New Zealand robins are unique in that they almost exclusively hoard insects that have been hunted and then paralyzed or killed and sometimes dismembered (Powlesland, 1980; Menzies and Burns, 2008). Robins then cache their prey in (and pilfer from) multiple caches containing small numbers of items throughout their territory (Powlesland, 1980; Menzies and Burns, 2008). This unusual combination of behavioral traits – caching highly perishable food items for only 1–3 days, storing food in multiple groups of small quantities, and a high rate of pilferage from conspecifics – could provide ideal conditions for advanced quantity discrimination to evolve.

Food-hoarding is employed by many avian species when faced with a surplus of food. However, in order for food-hoarding to be selectively advantageous, the hoarder must have a better chance of retrieving food stores than other animals (Andersson and Krebs, 1978; Smith and Reichman, 1984). An accurate memory for cache locations provides an important recovery advantage (Tomback, 1980; Sherry et al., 1981; Vander Wall, 1982; Kamil and Balda, 1985). Many scatter-hoarding species can accurately remember the locations of caches and outperform closely related, non-storing species in spatial memory tasks (Balda and Kamil, 1989; Bednekoff et al., 1997; Pravosudov and Clayton, 2002). This suggests that food-hoarding species often evolve cognitive specializations to remember large numbers of spatial coordinates associated with their cache sites. However, animals that create caches do not have exclusive access to their retrieval. Many species are known to pilfer caches made by other species (Burns and van Horik, 2007).

Accurate discrimination between sites containing variable numbers of items may be important when pilfering from caches; especially those located close together such as in the present study. Obtaining the highest available reward would make pilfering a more viable strategy if there is the possibility of aggressive repercussions from the owner of the cache. Robins preferentially select for cache sites containing more mealworms over those with fewer in the present study, as well as in Hunt et al. (2008) and Garland et al. (2012), strongly suggesting that the birds were actively avoiding these sites in favor of the sites containing the larger number of mealworms, which were selected at above-chance levels. Appropriate use of observational spatial memory (OSM) requires that an individual observe caching behavior from a distance in order to avoid alerting the cacher to the pilferer’s intentions. Such a distance would also minimize the visibility of minute size and volume differences in such items (which are often already minimized by the cacher dismembering and breaking larger prey into pieces), perhaps selecting for pilferage prioritizing based on cache size as a primary cue rather than prey size or volume. Making such observations would require an individual to maintain an accurate representation of the number of items observed being hidden in several different locations. Prioritizing the pilfering of the cache containing the largest quantity of prey would allow a pilferer to maximize energetic rewards gained during short temporal opportunities while the cacher is not in the vicinity. Because cache pilferers may face reprisals from food hoarders, the capacity to remember the number of items and locations where other animals have stored food could be selective advantageous.

The energy costs associated with food-hoarding mean that it is not surprising that some individuals adopt a “cheat” strategy by pilfering caches made by others. The result of the obvious incentive to steal is that strategies have evolved that allow individuals to conduct more accurate cache theft. After observing a conspecific caching, a pilferer can attempt to immediately steal or re-cache the food items. This is potentially dangerous however as the owner of the cache is likely to still be in the vicinity and may react aggressively if the thief is discovered. Because of this a less risky method is to observe and remember the location of a cache site and to return later when the owner is less likely to be around. This form of memory is referred to as OSM and has been identified as an important and advantageous cognitive ability for food pilfering (Bednekoff and Balda, 1996a,b; Scheid and Bugnyar, 2008). The ability to employ OSM when stealing cached food from others reduces the incidence of potentially dangerous aggressive encounters and may provide a means for subordinate individuals to compete indirectly for food without the need to physically displace dominant individuals.

Black-capped chickadees (*Parus atricapillus*) display excellent spatial memory in recovering their own caches (Baker et al., 1988; Hitchcock and Sherry, 1990). Despite this, black-capped chickadees showed no recovery benefit from observing another individual caching compared to recovering caches made in its absence (Baker et al., 1988; Hitchcock and Sherry, 1995). To date OSM has been demonstrated to varying degrees only in corvid species such as Pinyon jays (*Gymnorhinus cyanocephalus*), Mexican jays (*Aphelocoma ultramarina*), Clark’s nutcrackers (*Nucifraga columbiana*), scrub-jays (*Aphelocoma coerulescens*), jackdaws (*Corvus monedula*), and ravens (*C. corax*; Bednekoff and Balda, 1996a,b; Bednekoff et al., 1997; Bugnyar and Kotrschal, 2002; Scheid and Bugnyar, 2008). It has been hypothesized that OSM “could have evolved either as a consequence of extreme cache dependence, as a consequence of caching in flocks, or may have required the combination of these traits” (Bednekoff and Balda, 1996a, p. 824). Further research has produced mixed results in this area. Follow-up studies by Bednekoff and Balda (1996b) found that social Mexican jays (*A. ultramarina*) had a greater accuracy of recovery for caches that they had observed others making than more solitary Clark’s nutcrackers (*N. columbiana*). However, conversely, a similar study by Scheid and Bugnyar (2008) found that less social but more caching specialized ravens recovered other individual’s caches more accurately than social foraging, low-frequency caching jackdaw. In this instance the less social, but more cache-dependent species performed better than the socially cohesive species that cache only at low densities. Despite the differences in recovery ability both species were able to recover caches that they had observed another individual of the same species make. Black-capped chickadees, in contrast, gained no recovery benefit from observing caching behavior in a conspecific. Bednekoff and Balda concluded that enhanced spatial memory and social living are not both requisites for the evolution of OSM.

North Island robins (*Petroica longipes*) are not highly cache-dependent and rely on caching as an external mechanism for dealing with short-term temporal resource fluctuations (Menzies and Burns, 2008). When they do cache, North Island robins tend to maintain only a few active cache sites at any one time and will also reuse the same locations during subsequent caching bouts (Alexander et al., 2005). Robins are not social or flock foragers and are strictly monogamous (Higgins and Peter, 2002; Taylor et al., 2008), spending most of their lives in mate-pairs. Pairs usually form long-term associations and reside on permanent territories (Flack, 1976; Powlesland, 1980; Ardern et al., 1997; Armstrong et al., 2000). Although both members of the pair cooperate to raise young in the breeding season, males are competitively dominant to females and aggressively monopolize food sources year-round (Steer and Burns, 2008). Numerosity experiments involving a human demonstrator hiding mealworms (Hunt et al., 2008; Garland et al., 2012) showed that robins are capable of accurately locating food items that they have watched an individual of another species hide. This attentiveness to the actions of others suggested that New Zealand robins may be able to display OSM under experimental conditions. Like many animals that are endemic to isolated islands, New Zealand robins are fearless of humans (Alexander et al., 2005; Menzies and Burns, 2008). Their lack of anti-predatory behaviors toward humans facilitates the study of their cognitive abilities in the field. Wild birds can be approached and observed at very close distances (2–3 m).

New Zealand robins appear to possess a highly advanced quantity discrimination ability (Hunt et al., 2008; Garland et al., 2012). Wild birds were able to discriminate between hidden caches with unusually high accuracy far beyond a typical limit of four items in the absence of training. In violation of expectancy trials, they also searched for longer when some of the prey items they saw being cached were hidden from view before they were allowed to retrieve them. These results suggest that they could possess other sophisticated cognitive processes to enhance the likelihood of successful cache retrieval. While abstract numerical representation is yet uninvestigated in this species, the present experiment attempts to further investigate differential responses to quantitative discrimination of physical prey items under varying conditions, where stimuli such as visual access, time lag, and number of hiding places are all manipulated experimentally in a natural setting. It is hoped that this will provide a complementary example of an ecologically salient counterpart to similar, more abstract numerical processing tasks that lab-trained corvids have already proven to be capable of.

In this study, we sought to better understand OSM and prey quantity in a small passerine by conducting a series of experiments on a color-banded, wild population of North Island robins. Variable numbers of prey items were stored in a different number of artificial caches (2–4) in full view of subjects. Cache sites were then obscured from view for variable lengths of time (1–60 s). This experimental protocol was then repeated for different total numbers of stored prey (1–4). Results were then analyzed statistically to determine whether robins were capable of accurately choosing between multiple quantity comparisons that were obscured from view for variable lengths of time.

## Materials and Methods

This experiment was conducted at Zealandia, a 225 ha fragment of regenerating native bush located close to central Wellington, New Zealand (41° 18′ S, 174° 44′ E). Ten adult, male birds (18+ months of age) were used as subjects in all trials. All were uniquely color-banded for accurate identification.

**Figure 1:**
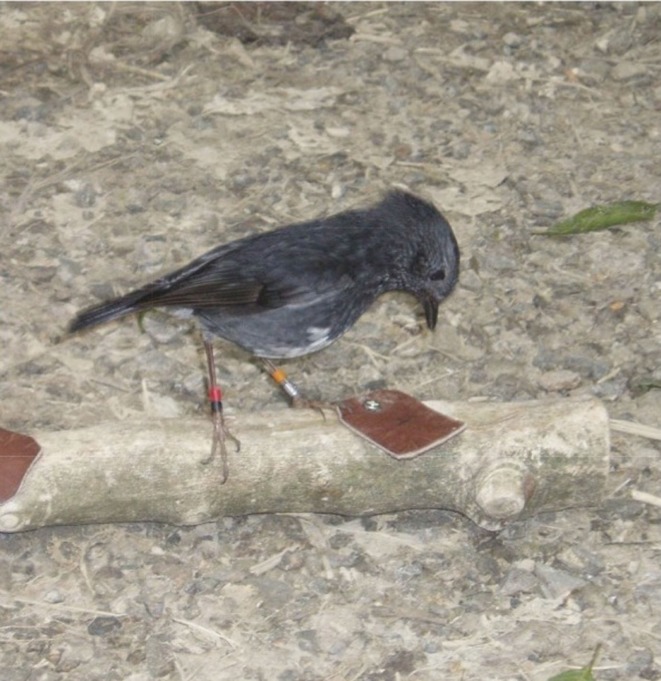
**A robin makes his choice by pulling a flap attached to the apparatus and retrieving the contents**.

This experiment was conducted using apparatuses similar to those used by Hunt et al. (2008). Each apparatus was constructed from a tree branch containing 2, 3, or 4 artificial depressions (manually drilled) that served as artificial cache sites (see Figure 1). Depressions were 3 cm long, 2 cm deep and were covered by a leather flap attached at one side by a screw swivel to conceal the contents from view. When wild birds forage naturally, they spend the majority of time searching for ground-dwelling invertebrates on the forest floor by turning-over dead leaves with their bill. Because leather flaps were similar in size, shape, and color to fallen leaves on the forest floor, all subjects learned to remove the flaps and retrieve the contents below with little or no training. Familiarization trials consisted of allowing birds to watch prey items being loaded into wells and covered. The birds were then permitted to access prey by allowing them to learn how to turn the leather flaps on the swivel. No comparisons were presented in familiarization trials, which served only to familiarize the bird with pulling the leather flap in the same manner as they overturn leaves. Once birds were able to pull flap to reveal contents, test trials commenced.

Three different variables were manipulated using a three factor, fully crossed design: quantity of mealworms (1v0, 1v2, 1v3), number of caches (2, 3, and 4), and retention interval (0, 10, and 60 s). This created a 3 × 3 × 3 design in which all 27 treatment combinations in the three conditions were repeated four times for each of the 10 color-marked individuals, yielding a total of 1080 trials.

For variable 1, the robins observed 1, 2, or 3 mealworms (*Tenebrio molitor* larvae) being placed in one artificial cache site. Additionally, a variable reward system was used in trials where 2 or 3 mealworms were hidden – an additional mealworm was placed in another cache site to test whether birds could discriminate between two non-zero values of reward. The number of artificial cache sites (variable 2) that robins could choose from was fixed-factor with three levels (2, 3, or 4 cache sites). This factor was included in the experiment to test whether the capacity of birds to choose the larger value declines as a function of the number of choice-locations present (i.e., cache sites). Retention interval (variable 3) or the period of time artificial prey was concealed from view was also fixed-factor with three levels: 0 s (i.e., the bird was allowed immediate access to the cache sites once the leather flaps had been closed), 10, and 60 s.

Memory traces decay over time and longer periods between caching and recovery or pilfering would be expected to lead to a corresponding decrease in success rate either through a reduction in memory accuracy or an increase in search errors. The retention intervals in these experiments were deliberately kept short as the study was designed as a test of OSM, rather than to determine how these memories decrease with time. In addition, the retention intervals in this study were constrained by the difficulties of working with a free-ranging population of birds. In the prolonged absence of visual stimulus (i.e., view of the mealworms or cache site) the robins lose interest and are more likely to abandon the experiment before a choice is made.

To homogenize potential learning effects, the order in which each treatment combination was conducted was assigned randomly. Trials were conducted between July and December 2010. The birds used in the experiment were located by spot-mapping along a series of tracks traversing the valley. Once the bird was located the experimental apparatus was placed on the track and trials began once the bird had approached within 2 m of the apparatus. The artificial cache sites were initially presented with the leather flaps open so the bird was able to see they were empty. Mealworms were then held up individually and displayed to the bird before being placed sequentially into the cache site (at a rate of approximately 5 s per item) and the leather flaps closed following Hunt et al. (2008). For trials where there was a variable reward, the order in which the sets of mealworms (i.e., larger number vs. smaller number) were placed in the cache sites was also randomized to control for potential order preferences. Once a choice was made, and prey was retrieved, the apparatus was removed from the experimental arena, giving birds the opportunity to retrieve from only a single cache.

In trials with a 0 s retention interval the experimenter then immediately stepped back 2 m and the robin was allowed to select and open one cache site. A cache was considered “selected” if the bird actively removed the leather flap from a well. The birds were allowed to retrieve any mealworms in the cache they had chosen, and not differentially reinforced for correct or incorrect responses outside of the differing quantities retrieved in the task itself. The same procedure was used in trials with retention intervals of 10 and 60 s, however after the leather flaps were closed the whole apparatus was covered with an opaque sheet. After the appropriate retention interval (10 or 60 s) the visual barrier (a cloth sheet) was removed and the experimenter stepped back and the trial proceeded as above.

A “successful” choice was defined as a trial in which the bird selected and removed the leather flap from the cache site containing the largest number of mealworms on the first attempt. A mean success rate was calculated as a percentage of successful choices for each treatment condition across the four replicates per individual bird, rendering individual birds as the unit of replication. If birds select cache sites at random, then the chance any particular site would be selected varies as a function of the total number of sites available. When there are two sites the likelihood of “success” by chance is 50%, compared to 33% when there are three sites presented and 25% when there are four sites.

To test whether birds performed above-chance expectations, in each treatment combination, the proportion of trials where birds chose the well with the highest number of mealworms was calculated for each bird. If birds chose sites randomly, the average of these values should be statistically indistinguishable from 1/*y*, where *y* is the number of cache sites available in each particular trial. To test whether birds performed above-chance expectations (i.e., the observed rates of “success” were unusually high), we conducted separate, single-sample *t*-tests for each treatment combination. In each test, we tested whether the average rates of “success” differed from randomized expectations (1/*y*). Separate tests were conducted for each treatment combination and *n* = 10 for all.

To test whether performances differed between retention intervals and the number of cache sites, separate linear mixed models were conducted for each condition. The number of cache sites and retention interval were considered fixed factors, each with three levels. Because all 10 birds were included in each treatment combination, “individual” was included in the model as a random factor. If the proportion of trials where the highest quantity of prey was chosen (i.e., success rate) was used as the dependent variable, a significant effect of number of cache sites would be observed even when birds were to choose cache sites at random. This effect due to varying number of cache sites arises mathematically from lower average chances of success in trials with more cache sites. To remove this confounding effect from analyses, the fraction of “successful” trials observed for each bird was subtracted from chance expectations (1/*y*) prior to analyses. All analyses were conducted in IBM Corp (2011) and data conformed to assumptions without transformation.

## Results

**Figure 2:**
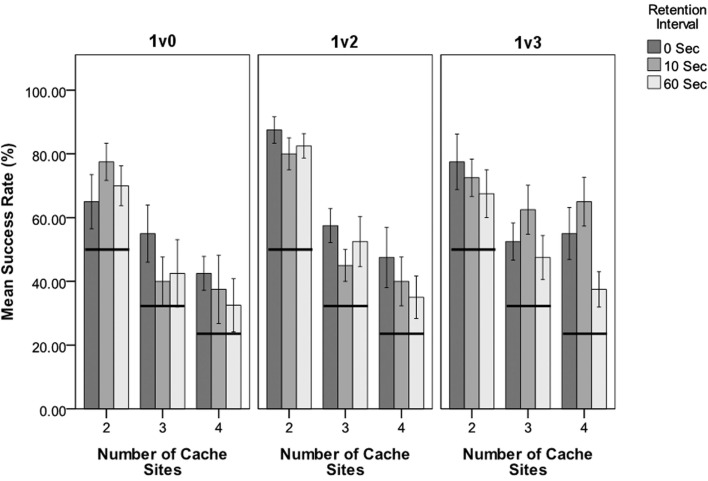
**The success rate for each combination of independent variables for the three conditions**. *Y*-axis shows the success rate as a percentage of “correct” choices (i.e., where the largest number of mealworms was selected). *X-*axis shows number of cache sites with bars grouped according to retention interval (0, 10, and 60 s). Indicates the percentage of successes expected by chance for each cache level. Error bars ± 1 standard error.

This experiment tested for non-random decision making with regards to selecting the larger presented number of mealworms. Each level of the two independent variables (number of cache sites and retention interval) was tested against chance expectation (Figure 2) for each of the three conditions. All different levels of number of cache sites (2, 3, or 4 sites) were significantly above-chance across all three conditions (*P* ≤ 0.021; Figure 2). All retention intervals (0, 10, and 60 s) were also significantly above-chance across the three conditions (*P* < 0.017 for all trials). This strongly suggests that North Island robins are capable of displaying OSM across at least short time intervals.

Robins chose the cache site containing the higher quantity of prey in 67% of trials. More specifically, across the 27 treatment combinations in the three conditions, the average “success” rate was higher than chance expectations in 18 trials (i.e., *P* < 0.05, Table 1). Two trials approached significance (0.05 < *P* < 0.10) and seven trials did not approach statistical significance (*P* > 0.10). “Success” rates were never below chance expectations. Additional *t*-tests were used to assess whether the robins selected the lower of the variable reward (i.e., the cache site containing only one mealworm) at a level that differed from chance. Trials where the cache site with only one mealworm was selected were significantly below chance level (*P* < 0.005; 1v2, *T* = −5.84; 1v3, *T* = −6.87). Robins did not preferentially select the smaller quantity of mealworms. Birds performed similarly regardless of retention interval and number of cache sites. Birds performed better (i.e., higher chances of success) when they were faced with fewer cache sites. A single-sample *t*-test across all 27 trails for whether the distribution of *t*-values listed in Table 1 differ significantly from a mean of zero shows significant difference (*t* = 8.194, df = 26, *P* < 0.001), providing evidence that robins consistently chose sites with more items at above-chance expectations across all trials. If robins chose cache sites at random, then the resulting *t*-values for each trial would form a distribution that would not differ from a mean of zero, whereas if they consistently choice sites with few items the mean *t*-value would be significantly negative.

**Table 1:** **Single-sample *t*-tests for all combinations of variables**.

Trial	Mealworms	Cache sites	Time	Mean	*T*	*P*
1	1v0	2	0	65.00	1.765	0.111
2	1v0	2	10	77.50	4.714	0.001
3	1v0	2	60	70.00	3.207	0.011
4	1v0	3	0	55.00	2.451	0.037
5	1v0	3	10	40.00	0.917	0.383
6	1v0	3	60	42.50	0.898	0.392
7	1v0	4	0	42.50	3.280	0.010
8	1v0	4	10	37.50	1.168	0.273
9	1v0	4	60	32.50	0.896	0.394
10	1v2	2	0	87.50	9.000	0.000
11	1v2	2	10	80.00	6.000	0.000
12	1v2	2	60	82.50	8.510	0.000
13	1v2	3	0	57.50	4.592	0.001
14	1v2	3	10	45.00	2.400	0.040
15	1v2	3	60	52.50	2.480	0.035
16	1v2	4	0	47.50	2.377	0.041
17	1v2	4	10	40.00	1.964	0.081
18	1v2	4	60	35.00	1.500	0.168
19	1v3	2	0	77.50	3.161	0.012
20	1v3	2	10	72.50	3.857	0.004
21	1v3	2	60	67.50	2.333	0.045
22	1v3	3	0	52.50	3.343	0.009
23	1v3	3	10	62.50	3.840	0.004
24	1v3	3	60	47.50	2.095	0.066
25	1v3	4	0	55.00	3.674	0.005
26	1v3	4	10	65.00	5.237	0.001
27	1v3	4	60	37.50	2.236	0.052

In trials where only a single mealworm was presented, neither the number of cache sites nor the retention interval were significant predictors of success. Both the interaction between number of cache sites and retention interval and the random factor “individual” were also non-significant (Table 2).

**Table 2:** **Results for general linear model analyses of variables 1v0 (top), 1v2 (middle), and 1v3 (bottom)**.

	SS	df	MS	*F*	*P*
**1v0 MEALWORMS**					
Individual	3090.278	9	343.364	0.405	0.903
Cache sites (Cs)	1335.556	2	667.778	0.865	0.438
Retention interval (Ri)	513.889	2	256.944	0.342	0.715
Cs × Ri	2069.444	4	517.361	0.766	0.554
**1v2 MEALWORMS**					
Individual	46013.611	1	46013.611	89.138	0.313
Cache sites (Cs)	4645.833	9	516.204	1.570	0.004
Retention interval (Ri)	5293.889	2	2646.944	7.573	0.189
Cs × Ri	1430.556	2	715.278	1.828	0.896
**1v3 MEALWORMS**					
Individual	50646.944	1	50646.944	135.337	0.567
Cache sites (Cs)	3368.056	9	374.228	0.964	0.637
Retention interval (Ri)	668.889	2	334.444	0.462	0.003
Cs × Ri	3930.556	2	1965.278	8.035	0.601

*SS, sums-of-squares; df, degrees of freedom; MS, mean squares; *F*, F-ratio; *P*, type-one error rates are shown*.

In trials where two mealworms were presented in one cache (1v2), number of cache sites was significant in determining the likelihood of a successful choice. Within these trials, those involving two cache sites had a higher likelihood of success compared to three or four cache sites (Figure 2). Retention interval, individual, and the interaction between number of cache sites and retention interval were all non-significant for this experiment (Table 2).

In trials where three mealworms were presented in one cache (1v3), number of cache sites was not a significant predictor of success, however retention interval was significant. Within these trials, those with a 60 s retention interval result in a lower rate of success than trials with a retention interval of either 0 or 10 s. Neither Individual nor the interaction term were significant (Table 2).

There were differences in success rate between the three quantity comparisons, suggesting that the number of mealworms offered as a reward affected the bird’s average success rate. In trials where the robins were offered only one mealworm there was a significantly lower success rate than in the other two experiments where the birds were offered two mealworms (*T* = −2.03, *P* = 0.46) or three mealworms (*T* = −2.37, *P* = 0.20). Trials with two or three mealworms did not differ significantly from each other (*T* = −0.32, *P* = 0.75). This may be indicative of a differing response to a higher number of null sets (empty caches) in these trials.

## Discussion

Results detailed here provide significant evidence that North Island robins are capable of utilizing OSM, at least over short time periods, when faced with variable cache numbers and prey quantities. Overall, they performed at above-chance expectation; however some treatment combinations were not above-chance (Table 1), but generally success decreased in a directional fashion as the complexity of the treatment increased. Treatments with a larger number of artificial cache sites would be expected to construe more of a memory challenge, as the birds must discriminate between a larger number of possible locations. Similarly, longer time frames are likely to reduce success rates as a result of temporal memory decay. When viewed in light of previous work on quantity discrimination in New Zealand robins (Hunt et al., 2008; Garland et al., 2012), it appears that this species may have evolved specialized abilities that facilitate the retrieval and pilferage of cached food.

To date OSM has not been demonstrated in a non-corvid avian species (Emery and Clayton, 2004). This study presents the first instance of another avian order with this cognitive ability. This finding is interesting, not only because it represents an incidence of parallel evolution of a cognitive trait, but also because New Zealand robins do not display many of the ecological traits that have been hypothesized as mechanisms behind the evolution of OSM in other species. Both sociality and high cache dependence have been posited as potential drivers in the evolution of OSM (Bednekoff and Balda, 1996a,b; Scheid and Bugnyar, 2008). New Zealand robins do not cache in high volumes (Alexander et al., 2005; Burns and van Horik, 2007; Menzies and Burns, 2008) and are not cache-dependent for winter survival (Menzies and Burns, 2008; Steer and Burns, 2008). Additionally robins are not highly social and so have limited opportunities to interact with conspecifics, and thus gain experience in social interactions. Despite not possessing either of these hypothetically important traits, robins show OSM over short time intervals, suggesting other pressures may have been influential in the evolution of this cognitive adaptation.

Intra-pair competition for resources may have been a driving force behind the evolution of OSM in North Island robins. Intra-pair resource competition in robins is intensive, especially during the winter (Steer and Burns, 2008; Menzies and Burns, 2010). Although they cooperate to raise young in the summer, intersexual relationships are decidedly antagonistic in winter (Alexander et al., 2005; Burns and Steer, 2006; Burns, 2009) with males being aggressive and competitively dominant over food resources. Individuals pilfer their mate’s caches (van Horik and Burns, 2007) and both sexes frequently re-cache both prey from their own or their mate’s caches (Steer and Burns, 2008).

Kamil and Gould (2008) note that there is a negative relationship between the cognitive demands of a cache recovery strategy and resistance of the strategy to competitors for the caches. Under conditions of high cache loss, increased cognitive abilities may be favored despite the large metabolic costs such cognitive abilities incur. The high level of cache loss and reciprocal cache pilferage in robins may have provided the necessary conditions for OSM to evolve, possibly driven by intraspecies sexual competition. Advances in OSM ability in one sex would likely be also conferred on the other sex over time, and an evolutionary “arms race” for better pilfering systems to reduce the impact of cache loss from pilfering could arise. Close social interactions between members of a pair may also provide the necessary social experiences for OSM to develop.

Given that New Zealand robins do not fulfill the hypothesized ethological confines for OSM: sociality and high levels of cache dependence, their memory abilities as displayed in this study appear to be more sophisticated than we had initially anticipated. This is surprising, as pilot studies had shown the birds performing at close to chance level when presented with three cache sites. Future work aimed at identifying the limits of their memory and quantitative ability would need to adopt longer retention intervals and larger numbers of cache sites. While a larger range of quantity comparisons investigated ratio and numerical distance effects in previous studies (Hunt et al., 2008; Garland et al., 2012), looking more in-depth at ratio and prey size/volume and the role it plays in decision making when more than two caches are present is also a salient aim for continued research with this species.

This study also included the use of a variable reward to test for not only OSM ability, but also to investigate the robins’ ability to make quantity judgments regarding pilfering activity. This is a novel feature of this study, as it shows that North Island robins are capable of sophisticated decision making regarding cache selection, even when required to rely on memory. The number of mealworms presented had an effect on the accuracy with which the robins were able to locate the hidden prey. Trials where the birds were only offered one mealworm had the lowest average success rate, whereas trials involving three prey items, had the highest average success rate. One thing to note is that in presenting a single quantity of worms (one worm) in the same number of caches, the nature of the task in these trials is somewhat different than the two conditions where two different quantities (1v2 and 1v3) were presented; the cognitive demand is on locating an item in an array of empty wells rather than discriminating quantities of prey. The difference in response may reflect the added complexity of including a higher number of null sets, or empty caches, present in trials where only a single mealworm was displayed. While a zero-like concept has been demonstrated in some animals (Pepperberg and Gordon, 2005; Merritt et al., 2009), no experimentation specifically focusing on null sets has been done with North Island robins to date. Without further research it is not possible to say definitively what the reason behind this number discrepancy is. Certainly, a number of additional factors could have played a role: increased motivation resulting from increased food reward, or a higher chance of momentary distraction influencing outcome when only one worm is dropped, for example. Both of these influences were minimized by halting trials if the bird appeared to not be watching the demonstration, only conducting a trial when the bird was less than 2 m away, and holding each worm in clear view prior to being placed in the artificial cache.

Hunt et al. (2008) conducted a series of experiments with robins that accounted for the potential confounding effects of the amount of time taken to fill each cache site with different numbers of prey items as well as for the volume of items in the trial. Neither of these factors were found to be significant in his study, meaning the robin’s ability to choose larger quantities is not related to either of these variables. While these factors are not ruled out as influencing the results of the present study, there is no indication that their influence should differ between these studies, as the methods and nature of the prey retrieval task are essentially the same.

Trials with one vs. two mealworms comprised the only group of trials where there was a significant difference in success rate between the number of sites. In this instance it was the two-site trials that had the higher success rate (Figure 2), i.e., the less complex treatments. However trials involving three or four cache sites still produced above-chance success rates. The number of cache sites selected for these experiments was based on the average number of individual cache sites that a robin might generally maintain at any one time, which has been observed as one to three different cache sites (van Horik and Burns, 2007) within view of an observing experimenter, when presented with a overabundance of prey. The fact that number of cache sites was non-significant in two of the three conditions here shows that robins are capable of distinguishing between a larger number of locations than the maximum of four sites used in this study. This suggests that robin’s possess the ability to track more locations than they may typically utilize for their own caching needs. Increased memory load can cause an increase in interference in memory retrieval. Being able to recall more separate locations than the robins require for their own caching needs would be a useful memory component for OSM. It would allow an individual to monitor the locations of caches belonging to others without the risk of displacing memories for their own cache sites.

The effect of retention interval is difficult to interpret from this study as retention interval was significant only in trials that offered a maximum of three mealworm prey items in a single cache. Within this condition, the longest 60 s trials had a lower success rate than the 0 or 10 s trials, which did not differ significantly from each other. While lower success rates for the longest trials may suggest that accuracy decreases somewhat over a period of 60 s, this still appears to be within the memory capabilities of North Island robins. It should be noted however that the 0 s trials were also methodologically different from the longer retention intervals as they did not involve the cache sites being occluded from view. In this respect the 0 s trials were not a test of memory and so are not directly comparable to the other retention intervals. A decrease in accuracy over comparatively short periods should be predicted by current knowledge of robin caching behavior. Cache recovery by robins is usually on the same day that the cache is created, and always within 3 days (Powlesland, 1980). Pilfering on the other hand usually occurs over shorter intervals of less than 30 min (van Horik and Burns, 2007), and often within a few minutes of caches being created.

The retention intervals used in this study are significantly shorter than those of the corvid and parid studies that currently make up the majority of the literature on OSM. Parid studies used retention intervals ranging from 6 min to 2 h (Baker et al., 1988), while corvid studies covered a wide range of intervals, from 5 min to 7 days (Bednekoff and Balda, 1996a,b; Bugnyar and Kotrschal, 2002). However these species are predominantly long-term hoarders that rely on cached food for significant proportions of their winter energy requirements. The shorter retention intervals used in this study were more ecologically relevant for robins given the time periods over which most of their cache recovery and pilfering activities take place. It should be noted however that Scheid and Bugnyar (2008) also used a 1 min retention interval for ravens and jackdaws. Of the current literature in the observational memory area, Scheid and Bugnyar’s study is methodologically closest to the study presented here. Short retention intervals were used, along with small numbers of artificial cache sites (between 2 and 10) and the birds observed a human experimenter hiding food items rather than a conspecific.

Bugnyar and Kotrschal (2002) also noted that the ravens used in their study began pilfering attempts between 1 and 2 min after watching the caching event. This suggests that even in ravens, a species shown to possess accurate and flexible OSM abilities (Heinrich and Pepper, 1998; Bugnyar and Heinrich, 2006; Scheid and Bugnyar, 2008), pilfering is still conducted soon after witnessing caching. In situations where there is a large amount of food available, pilfering soon after the caching event may be advantageous as it is likely that, after creating one cache, the storer will continue to create more caches in different locations for as long as the food source persists. During this period of caching the individual may be distracted from monitoring the first cache it created, thereby allowing a window where pilfering can safely occur. Robins cache in a highly complex temperate rainforest, as opposed to in more open environments. This provides many opportunities for a potential thief to be out of sight and thus able to re-cache items with a reduced risk of being noticed. This would be expected to reduce the incentive to develop longer-term pilfering strategies as short-term approaches may be equally effective, without the need for more advanced cognitive abilities.

Possibly because robins do not risk high levels of cache theft from individuals (either con- or hetero-specific) other than their mates, and they benefit genetically from having a healthy mate, this species may be able to tolerate higher levels of pilferage than flock foraging species where kinship is low and there is no direct benefit from cache loss. The presence of other individuals (either the study bird’s mate or another individual) was not recorded in this study and it is possible that this may have impacted on the birds’ cache retrieval decisions.

Robins provide a new avian model: small passerines that nevertheless are capable of displaying sophisticated cognitive abilities. While many of the robins’ cognitive processes may not be as complex as those displayed by corvids or parrots, they may provide an interesting intermediate. Studies on robins could be used to shed light on the conditions necessary for these advanced cognitive abilities to evolve. New Zealand robins do not display either high levels of sociality or cache dependence, the two traits hypothesized to be mechanisms leading to OSM evolution in corvids. This suggests that there are alternative pressures that could drive the evolution and development of this trait, at least in North Island robins. Intensive intra-pair competition for resources, characterized by high levels of reciprocal cache theft can be proposed as a possible mechanism leading to advanced cognitive traits that improve pilfering strategies. Comparatively little is known about the extent to which non-human animals are capable of identifying inequalities that involve more than two quantities of items. The results from this study indicate that New Zealand robins appear to successfully choose a larger quantity of mealworms when confronted with multiple possible obscured caches and delays in access, but that the accuracy with which they do so is not necessarily related to each of these features in a predictable linear way. These initial findings lay the groundwork for continued research into the myriad of influences that may play a role in avian cognition and cache strategy for this small songbird.

## Conflict of Interest Statement

The authors declare that the research was conducted in the absence of any commercial or financial relationships that could be construed as a potential conflict of interest.
